# Clinical Characteristics and Predictive Features of Neonates With Treatment‐Needed Retinopathy of Prematurity: A Retrospective Cohort Study

**DOI:** 10.1002/hsr2.70892

**Published:** 2025-06-11

**Authors:** Nasser Shoeibi, Fereshteh Raoufi, Majid Abrishami, Seyedeh Maryam Hoseini, Mohammad‐Reza Ansari‐Astaneh, Mojtaba Abrishami, Ghodsieh Zamani, Elham Bakhtiari, Razieh Farrahi, Bahareh Gharib, Fatemeh Neghabi, Nasibe Zare Shahneh, Mehrdad Motamed Shariati

**Affiliations:** ^1^ Eye Research Center Mashhad University of Medical Sciences Mashhad Iran

**Keywords:** NICU hospitalization, premature, retinopathy of prematurity, risk factor, treatment

## Abstract

**Background:**

Several variables have been identified as risk factors for retinopathy of prematurity (ROP). This study evaluated the risk factors associated with the increased need for treatment in infants with ROP.

**Methods:**

In this longitudinal study, the medical records of premature infants referred to the ROP clinic of a tertiary referral center in northeast Iran between July 2014 and June 2022 were reviewed. Probable neonatal and maternal risk factors for the need for treatment were analyzed.

**Results:**

Of the 9692 referred neonates, 4634 (47.8%) were diagnosed with any stage of ROP. The majority (53%, *n* = 4141) were male. The mean gestational age (GA) was 32.71 ± 2.52 weeks, and the mean birth weight (BW) was 1812 ± 501 g. 427 (4.4%) of 9692 referred infants were treated. In multivariate logistic regression analysis, lower gestational age (OR, 0.65; 95% CI, 0.53–0.79; *p* < 0.001), lower birth weight (OR, 0.8; 95% CI, 0.68–0.93; *p* < 0.001), and longer length of stay in neonatal intensive care unit (NICU) (OR, 1.59; 95% CI, 1.55–1.63; *p* < 0.001) were significantly associated with the need for treatment.

**Conclusion:**

We showed that lower birth weight, lower gestational age, and more extended NICU hospitalization were significant predictors of the development of ROP and treatment‐needed ROP. Besides, the prevalence of ROP was significantly lower among patients with maternal gestational hypertension.

## 1 Introduction

Retinopathy of prematurity (ROP) is defined by abnormal retinal blood vessel development in premature infants, which causes visual impairment at an early age [1, 2]. During the past few decades, much progress in neonatal care has decreased the mortality of premature infants, and as a result, the survival rate of patients with ROP has increased [3, 4]. Short gestation (< 30 weeks), low birth weight (LBW) (under 1500 g), and hyperoxia are among the main factors that increase the risk of ROP [5, 6, 7]. ROP risk factors include multiple births, neonatal sepsis, intraventricular bleeding, low Apgar score, lung diseases, infection, prolonged mechanical ventilation, heart disease, blood transfusions, and male gender [8, 9, 10, 11, 12]. Previous literature reported that some maternal and neonatal underlying diseases can play a role in the occurrence and development of ROP [13, 14, 15, 16, 17]. Depending on socioeconomic status, income level, preterm birth rates, and neonatal care, the incidence of ROP varies in different regions worldwide, from 4.2% to 80%. There have been numerous epidemiologic studies conducted to find ROP risk factors. However, predicting high‐risk infants is still difficult [18, 19]. Zarei et al. in 2019 showed that the prevalence of ROP was 27.3% among screened infants in Tehran, the capital city of Iran [20]. Azami et al. investigated the prevalence and risk factors of ROP in Iran through a systematic review and meta‐analysis. They reported that the prevalence of ROP is 23.5% in Iran. Small gestational age, low birth weight, septicemia, intraventricular hemorrhage, and respiratory distress syndrome were the most significant factors [21].

According to studies conducted in Iran, the incidence of ROP was relatively high in this country [1, 22]. There are discrepancies among the studies regarding the prevalence of treatment‐needed ROP neonates. The small sample size was the main limitation of these studies, so we attempted to collect comprehensive information about the risk factors of ROP, the number of newborn examinations, and treated infants from 2014 to 2022.

This study aimed to identify the role of maternal and neonatal factors in the incidence and progression of ROP in patients referred to the ROP clinic of a tertiary referral center in northeast Iran. We also investigated the risk factors that increase the need for treatment in these patients.

## 2 Methods

In this longitudinal retrospective study, we reviewed the medical records of all premature infants admitted to the ROP clinic of Khatam Eye Hospital of Mashhad, Iran, between 2014 and 2022. This center is the only organized facility for ROP screening in northeast Iran and covers more than 5 million people. All infants who indicated screening examination for ROP were included in the study. Data were collected from the medical records of patients, including sex, gestational age (GA), birth weight (BW), single/multiple gestations, neonatal intensive care unit (NICU) stay time, loss of one twin, maternal Pre‐eclampsia, and maternal hypertension. The mother's level of education was also considered as a variable of the socioeconomic level of the family.

Infants with missing information regarding the occurrence of ROP, follow‐up time, and need for treatment were excluded.

This study was approved by the institutional ethics committee (IR.MUMS.MEDICAL.REC.1400.308), and informed consent was obtained from the parents or guardians of preterm neonates before starting the first screening examination. Also, informed consent for publication of the results of examination for research purposes was obtained from the parents or guardians of preterm neonates.

### 2.1 Screening Protocol

According to the Iranian national guidelines, infants with a birth weight (BW) of ≤ 2000 g or GA of < 34 weeks, as well as infants with a BW > 2000 g or GA ≥ 34 weeks who had a history of unstable systemic condition were referred for ROP screening. The RetCam imaging system is used at this facility to photograph all preterm infants who require a retinal examination by national criteria following dilation of the pupils. Images and demographic information about patients, such as gestational age and birth weight were stored in the registry system.

Based on the International Classification of Retinopathy of Prematurity, each patient was categorized according to the most advanced stage of ROP in eyes discovered during follow‐up examination. The Early Treatment for Retinopathy of Prematurity randomized trial's classification of patients as having type 1 prethreshold ROP or not also determined as the treatment‐needed criteria [23].

### 2.2 Treatment Protocol

This facility offers bevacizumab injections (0.625 mg in 0.025 mL) intravitreally and laser photocoagulation of avascular retinal regions as two therapeutic options for infants with treatment‐needed stages of ROP. The severity of the illness, the infant's current age, the gestational age, and the attending physician's suggestion are crucial factors to consider when choosing a course of therapy. The patients' advanced age and the existence of vascular loops and shunt vessels at the boundary between vascular and avascular retinal zones are the influential factors in choosing laser photocoagulation as the treatment option. In patients with a history of laser photocoagulation, intravitreal bevacizumab (IVB) injection is not thought to be a viable alternative for rescue therapy in recurrent disease.

### 2.3 Statistical Analysis

The collected data were analyzed using SPSS software version 26 (SPSS Inc., Chicago, IL, USA). Frequency was used to describe qualitative variables, while mean and standard deviation (SD) were used for quantitative data. Chi‐square and t‐tests were used to compare the features between the two groups of ROP and no‐ROP. Multivariable Logistic regression analysis was performed to determine the risk factors associated with the incidence of ROP as well as the need for treatment. The odds ratio (OR) and 95% confidence interval (CI) for each risk factor were determined, and a *p* value less than 0.05 was considered statistically significant. In the presence of a *p* value less than 0.05, OR values greater than 1.00 were considered risk‐increasing factors, while OR values less than 1.00 were considered risk‐reducing factors.

## 3 Results

### 3.1 General Characteristics of the Patients

Generally, 9692 infants were screened for ROP during the 8‐year study period, of whom 4634 (47.8%) were diagnosed with ROP at any stage. The median number of visits for participants was 3 (inter‐quartile range: 2–4) and the median number of follow‐up days was 29 days (inter‐quartile range: 14–58 days).

Among the study population, the majority (53%, *n* = 4141) were male. The mean GA of the patients was 32.71 ± 2.52 (ranging, 25–38) weeks, and the mean birth weight (BW) was 1812 ± 501.4 g (range, 560–4000 g). The mean duration of the infant's NICU hospitalization was 12.80 ± 12.90 (0–96) days.

In this study, we evaluated the factors affecting the development of ROP and investigated the features associated with the need for treatment.

The maternal and neonatal risk factors associated with ROP development are presented in Table 1.

**Table 1:** Comparing the maternal and neonatal features in the two subgroups of neonates, with ROP and no‐ROP.

	No ROP	ROP	*p* value
Gestational age (*n* = 9671) (Weeks)	33.52 ± 2.13 (*n* = 5028)	31.60 ± 2.30 (*n* = 4624)	**< 0.001** *
Birth weight (*n* = 9682) (Grams)	1920.24 ± 459.35 (*n* = 3228)	1653.32 ± 450.02 (*n* = 4632)	**< 0.001** *
Length of NICU stay (*n* = 9687)	9.48 ± 10.23 (*n* = 3231)	16.50 ± 14.36 (*n* = 4634)	**< 0.001** *
Gender			
Male (*n* = 4141)	1690 (52.3%)	2451 (52.9%)	0.599
Female (*n* = 3725)	1542 (47.7%)	2183 (47.1%)	
Parity (*n* = 9550)			
Singleton (*n* = 6366)	6072	294	0.318
Multiple (*n* = 3182)	3052	132	
fetal death in multiple gestation			
Yes (*n* = 124)	32(1%)	92 (2%)	**< 0.001** *
No (*n* = 7742)	3200 (99%)	4542 (98%)	
Maternal hypertension			
Yes (*n* = 287)	170 (5.3%)	117 (2.5%)	**< 0.001** *
No (*n* = 7579)	3062 (94.7%)	4517 (97.5%)	
Maternal Pre‐eclampsia			
Yes (*n* = 15)	10 (0.3%)	5 (0.1%)	**0.044** *
No (*n* = 7851)	3222 (99.7%)	4629 (99.9%)	
Previous history of abortion			
Yes (*n* = 553)	252 (7.8%)	301 (6.5%)	
No (*n* = 7313)	2980 (92.2%)	4333 (93.5%)	**0.026** *

* Considered statistically significant.

### 3.2 Neonatal Risk Factors for the Development of ROP

Among neonatal risk factors, GA, BW, and length of NICU stay were significantly different between infants with ROP and those without ROP.

### 3.3 Maternal Risk Factors for the Development of ROP

Maternal risk factors, such as fetal death in multiple gestation, hypertension, presence of pre‐eclampsia, and previous history of abortion, were significantly different between infants who developed ROP and those who did not.

Logit regression analysis was performed to determine variables predicting the development of ROP (Table 2).

**Table 2:** Risk factors associated with the development of ROP.

	ROP at any stage, *n* = 4634		
Risk factors	Odds ratio	95% CI	*p* value
Gestational age	0.70	0.68–0.73	**< 0.001** *
Birth weight	0.79	0.74–0.89	**< 0.001** *
NICU_Time	1.01	1.01–1.02	**0.009** *
Gender	1.05	0.95–1.16	0.239
Multiple gestations	1.02	0.92–1.12	0.232
Loss of one twin	1.22	0.77–1.23	0.344
Maternal HTN	0.81	0.77–0.85	**< 0.001** *
Maternal Pre‐eclampsia	0.93	0.83–1.35	0.091
Previous abortion history	1.30	1.07–1.57	**< 0.001** *
Mother education	0.91	0.82–1.35	0.323

* Considered statistically significant.

### 3.4 Treatment‐Needed Patients

Overall, among the 9692 preterm infants screened for ROP, 427 (4.4%) underwent treatment, with 392 patients (91.8%) receiving intravitreal bevacizumab injection, 33 patients (7.72%) undergoing retinal laser photocoagulation, and 2 patients (0.46%) undergoing pars plana vitrectomy. The remaining infants did not require any clinical intervention during the follow‐up period. A total of 154 infants screened for ROP were treated at the first visit in our study, of which 150 (97.4%) received intravitreal bevacizumab injection, 3 (1.9%) were treated with laser, and 1 (0.6%) underwent vitrectomy. All patients were followed up till complete retinal vascularization. Twenty‐three patients (44 eyes) experienced the recurrent disease. IVB (11 eyes), laser photocoagulation (23 eyes), pars plana vitrectomy (1 eye), and scleral buckle surgery (1 eye) were the rescue procedures.

### 3.5 Risk Factors for ROP That Were Associated With the Need for Therapy

Logit regression analysis was performed to determine variables predicting the need for therapy in patients with ROP. Detailed information is provided in Table 3.

**Table 3:** Risk factors associated with the treatment‐needed ROP.

	Treatment‐needed patients, *n* = 427		
	Odds ratio	95% CI	*p* value
Gestational age	0.65	0.53–0.79	**< 0.001** *
Birth weight	0.8	0.68–0.93	**< 0.001** *
NICU_Time	1.59	1.55–1.63	**< 0.001** *
Gender	0.96	0.794–1.162	0.68
Multiple gestations	0.86	0.705–1.065	0.17
Loss of one twin	0.85	0.46–1.55	0.59
Maternal HTN	1.67	0.90–3.08	0.10
Maternal pre‐eclampsia	1.64	0.231–11.713	0.62
Previous abortion history	0.74	0.568–1.295	0.23
Mother education	0.83	0.638–1.079	0.16

* Considered statistically significant.

The analyses identified lower gestational age (OR, 0.65; 95% CI, 0.53–0.79; *p* < 0.001), lower birth weight (OR, 0.8; 95% CI, 0.68–0.93; *p* < 0.001), longer length of stay in NICU (OR, 1.59; 95% CI, 1.55–1.63; *p* < 0.001), and use of fertility drugs for getting pregnant (OR, 1.68; 95% CI, 1.17–2.41; *p* = 0.005) as significant risk factors that were associated with the need for therapy.

## 4 Discussion

This study aimed to determine the risk factors for ROP development and to assess the potential risk factors associated with the need for treatment in infants with ROP. Gestational age, birth weight, and duration of NICU hospitalization were the significant predictive factors for treatment among preterm infants with ROP.

In our study, the overall incidence of any stage of ROP in premature infants was 47.8% (4634 of 9692). This finding is comparable with the retrospective study by Yang et al. [24], in which the incidence of ROP was approximately 45.8% (99 of 216) in very‐low‐birth‐weight infants, and approximately 33.9% (204 of 602) in the study by Freitas et al. [25], who also included infants with a birth weight of more than 1500 g or a gestational age of more than 32 weeks in their study. The incidence of ROP in infants with a birth weight of ≤ 1500 g has been reported to be between 33% and 45% in developing countries [11, 26, 27]. However, the incidence of ROP varies among countries based on socioeconomic status, the quality of neonatal care provided, and the inclusion criteria for screening protocols [28]. Improving the NICU care quality in developing countries leads to a higher survival rate of preterm infants and increases the prevalence of ROP.

Consistent with previous studies, we found that neonatal risk factors, including gestational age, birth weight, length of NICU stay, and maternal risk factors, including fetal death in multiple gestations, and previous history of abortion, were associated with the development of ROP. Recently, the results of a meta‐analysis showed that the prevalence of ROP was 23.5% in Iran. They concluded that low gestational age, low birth weight, respiratory distress syndrome, septicemia, intraventricular hemorrhage, duration and frequency of oxygen therapy, blood transfusion, and phototherapy were significant risk factors for the development of ROP [21].

In this study, we found that the prevalence of maternal hypertension and pre‐eclampsia was significantly lower in the ROP group compared to no‐ROP. Yu et al. [29] found that pre‐eclampsia was associated with a reduced risk of ROP, whereas, gestational hypertension was not significantly associated with ROP at early or late preterm births. Many studies have demonstrated that pre‐eclampsia is a protective factor for ROP in premature infants [25, 26, 27, 30]. pre‐eclampsia is caused mainly by an increase in the circulating soluble form of vascular endothelial growth factor (VEGF) receptor‐1, or Flt‐1. This receptor may bind both VEGF and placental growth factor (PGF). ROP pathogenesis is significantly influenced by VEGF signaling, and anti‐VEGF therapy has been demonstrated to be highly beneficial in treating ROP [31]. It is challenging to investigate an independent relationship between maternal pre‐eclampsia and ROP, because possible biases, including small GA and low birth weight, may contribute to both pre‐eclampsia and ROP.

Numerous prior studies have identified low birth weight and gestational age as significant risk factors in developing ROP [29, 32, 33, 34, 35]. These findings were consistent with our results. Moreover, they also found that a long duration of oxygen therapy is associated with ROP progression in more mature infants [10, 36].

Lin et al. [37] reported that low gestational age, low birth weight, male sex, respiratory complications, and length of stay in NICU, were associated with an increased incidence of ROP. The results of our study are similar to those of Lin et al. but sex was not an independent risk factor for the development of ROP.

Treatment was generally performed in 427 (4.4%) of the 9692 patients in the current study. Therefore, we attempted to detect the risk factors related to the need for treatment in ROP infants. According to the results of multiple logistic regression in our study, among neonatal risk factors for ROP infants, gestational age, birth weight, and length of NICU stay were the most predictive factors for treatment among preterm infants with ROP.

Bas et al. [26] reported that some infants with BW > 1500 g and GA > 32 weeks, which are defined as bigger and mature babies, are at risk of developing severe ROP that requires treatment. In contrast, Gerull et al. [38] found that most ROP infants with GA < 28 weeks required ROP therapy. Hwang et al. [39] reported that the incidence of ROP was 11.5% (231 of 2,009). They reported that 28.1% (193 of 688) of ROP infants with GA < 28 weeks and 26.5% (186 of 701) with BW < 1000 g required treatment. According to Freitas et al. [25], low BW (< 1000 g) and the presence of pulmonary diseases are significant risk factors for type 1 pre‐threshold ROP with indications for ROP treatment. Recently, a few studies have shown that an extended stay in the NICU is accompanied by a higher risk of ROP development [37, 40]; however, to our knowledge, the association of duration of NICU hospitalization with treatment‐needing ROP cases has not yet been studied.

Although this study, which included nearly 10,000 patients, is one of the most extensive studies conducted in Iran, it has some limitations. The retrospective design of this study is one of its limitations. Missing data and some errors may have occurred in the patient documentation. We included infants born within an 8‐year period, which could be another limitation of our study because of the improvements in the neonatal care system during this long period. Besides, the risk factors associated with the treatment failure had not been investigated in this study.

The main strength of the study was its high number of participants. Also, this study was according to the national screening protocol for ROP neonates. We investigated numerous neonatal and maternal factors including the mother's education, in all participants regarding whether they influence the ROP development and the prevalence of the treatment‐needed patients.

## 5 Conclusion

In this retrospective cohort study, we investigated the clinical characteristics and predictive features of neonates diagnosed with ROP, with a particular focus on those requiring treatment. The overall incidence of ROP among the premature infants included in this study was 47.8%. This significant incidence underlines the importance of vigilant screening and timely intervention for at‐risk infants to prevent the potential sight‐threatening consequences of severe ROP. Among the strongest predictors, low gestational age, low birth weight, prolonged stay in the NICU, and a maternal history of abortion were significantly associated with increased risk of developing ROP. Notably, our analysis revealed that of the neonates diagnosed with ROP, only 4.4% required treatment, indicating that the majority of cases were self‐limiting or did not progress to severe stages. Importantly, the study identified three key factors—low gestational age, low birth weight, and prolonged NICU stay—as significant predictors for those neonates who required treatment for ROP.

## Author Contributions

**Nasser Shoeibi:** conceptualization, project administration, writing – review and editing, supervision. **Fereshteh Raoufi:** data curation, investigation. **Majid Abrishami:** data curation, investigation, project administration. **Seyedeh Maryam Hoseini:** data curation, supervision, investigation. **Mohammad‐Reza Ansari‐Astaneh:** data curation, investigation. **Mojtaba Abrishami:** data curation, investigation. **Ghodsieh Zamani:** data curation, investigation. **Elham Bakhtiari:** investigation, formal analysis, methodology. **Razieh Farrahi:** data curation. **Bahareh Gharib:** data curation. **Fatemeh Neghabi:** data curation. **Nasibe Zare Shahneh:** data curation. **Mehrdad Motamed Shariati:** data curation, writing – review and editing, writing – original draft, investigation, formal analysis, supervision.

## Conflicts of Interest

The authors declare no conflicts of interest.

## Transparency Statement

The lead author Mehrdad Motamed Shariati affirms that this manuscript is an honest, accurate, and transparent account of the study being reported; that no important aspects of the study have been omitted; and that any discrepancies from the study as planned (and, if relevant, registered) have been explained.

## Data Availability

All data presented in the result but the raw data that support the finding of this study are available from the corresponding author on reasonable request.
